# Obituary

**Published:** 1869-12

**Authors:** 


### Obituary.

Died in Geneva, N. Y., Dec. 3d, Hazard Arnold Potter, M. D. He was born in Potter township, Gates county, Dec. 21st. 1811, graduated in medicine at Bowdoin College in 1835, practiced his profession a short time in Rhode Island, thence went to his native place, where he practiced till 1853, when he removed to Geneva. He was, during the war, Surgeon to the 50th N. Y. Vols. He was an energetic worker, attained reputation as a surgeon for his successful trephining of the vertebral column, operations for ovariotomy, and method of amputating at the hip-joint. During the last four years he has been a prominent advocate of temperance, which cause he sustained with characteristic zeal. The immediate occasion of his death was inflammation of the lungs.

